# The effects of theatre-based vocal empowerment on young Egyptian women’s vocal and language characteristics

**DOI:** 10.1371/journal.pone.0261294

**Published:** 2021-12-31

**Authors:** Sarah Fahmy, Pui-Fong Kan, Jen Walentas Lewon

**Affiliations:** 1 Department of Theatre and Dance, University of Colorado Boulder, Boulder, Colorado, United States of America; 2 Department of Speech, Language, & Hearing Sciences, University of Colorado Boulder, Boulder, Colorado, United States of America; University of Ioannina: Panepistemio Ioanninon, GREECE

## Abstract

This study investigates the impact of a theatre-based vocal empowerment program on the vocal and language characteristics and the self-perceptions of young bilingual Egyptian women. The program used applied theatre, a dramatic practice that promotes civic action by utilizing improvisational techniques to engage participants in exploring solutions to self-identified community concerns. These techniques supported participants’ pursuit of vocal empowerment: the ability to comfortably express their intended content with a clear audible voice, accompanied by the belief that what they had to say was worthwhile. The program was implemented in Alexandria and Aswan, two Egyptian cities in different regions of the country, with distinct socio-economic profiles. Thirty-six young women from Aswan and nineteen from Alexandria participated. The program was facilitated in Arabic, for 90 minutes per day over twelve consecutive days in 2018. Participants in both groups spoke Arabic as a home language and studied English in school settings but differed in their educational experiences and English proficiency. The vocal and language characteristics of each participant were tested in Arabic and English pre- and post- program using a spontaneous speech task and a reading aloud task. Their self-perceptions were evaluated through a vocal self-perception survey. Results indicated that participants responded differently in each city. In Alexandria, participants showed significant improvement in language skills (e.g., mean length of utterance). In contrast, participants in Aswan showed a significant change in fundamental frequency. Overall, the self-surveys indicated that all participants experienced an increased sense of confidence, a stronger belief in self-authorship, and an increased desire to voice their opinions clearly in public; however, there were subtle differences between the groups. In analyzing these results, we conclude that to design effective vocal empowerment outreach programs internationally, it is necessary to consider participants’ cultural backgrounds, language diversity, and socio-economic status.

## Introduction

Young women who have an increased understanding and awareness of their voices, have an increased ability to positively impact their self-advocacy to express themselves, and contribute to their communities [1]. A young woman’s voice quality has been linked to her sense of identity, perceived dominance, and personality, and has been an integral focus within women empowerment initiatives [2, 3]. Since theatre is viewed as a particularly effective tool to expand a person’s vocal range, and support participants’ self-advocacy [4], in the current study, we investigate the impact of a theatre-based vocal empowerment program on young bilingual Egyptian women’s vocal and language production characteristics, and examine participants self-perceptions of their own voices. Empowering women’s voices through theatre could potentially be a cost-effective and sustainable way to increase gender equality and women’s civic engagement in the economic and political spheres, as it only requires human willpower, and guided facilitation [5]. The program was conducted with young women, residents of Alexandria and Aswan, Egypt. These two major cities lie in different geographical regions of the country and demonstrate distinct socio-economic profiles; the residents of the two cities vary in their exposure to globalization, and in their use of Arabic dialects (L1) and English (L2) in daily life. These are important considerations to assess the impact of a theatre-based vocal empowerment program.

The program used in this study is an adaptation of the SPEAK Vocal Empowerment Curriculum, initially developed by Osnes and Hackett in partnership with Guatemalan non-profit, *Starfish*: *MAIA Impact*, a Guatemalan all-girls school serving Indigenous youth [1]. Vocal empowerment is a person’s ability to comfortably express their intended content, with a clear audible voice. It is the belief that “what you say is worthwhile, your voice belongs to you, and that you have the right for self-authorship” [5]. The curriculum used in this study has previously been proven to enable young women to achieve a greater sense of self-authorship, ownership of their voices, and confidence in their bodies through the practice of applied theatre [1].

Applied theatre is an umbrella term used to describe theatre practices that intentionally and creatively foster an environment for participants to practice using their voices to speak up about community issues they care about. It is an inclusive, flexible participant-led artistic practice that caters to the needs of the community [6]. In Egypt, applied theatre has been facilitated by non-governmental organizations, commissioned by UN Women, and by local independent Egyptian theatre companies nationwide. Nevertheless, the art form itself is relatively unfamiliar. The workshops that have been implemented were conducted with a variety of community members, including women, people with disabilities, refugees, prisoners, sex workers, and young garbage pickers. They focused on a variety of issues including: second language learning, living with disabilities, sexual harassment, and female genital mutilation [7–12]. Through improvisation, voice exercises, and the embodied exploration of societal issues, these workshops emphasized both community building and the importance of listening to personal narratives to empower participants. These previous efforts indicated that applied theatre was well-positioned to impact the vocal and language characteristics of the young Egyptian women who participated in this study.

Very few studies have directly examined such vocal empowerment programs and their effect on women’s vocal and language characteristics [1]. To our best knowledge, no studies have documented measurable outcomes of applied theatre for young women’s vocal empowerment in Egypt. Nor are there studies on the differences in vocal empowerment between different levels of bilinguals in the country or comparing the self-perception of young women in different parts of the country. The facilitation and outcomes of this study may be beneficial to scholars in several fields including but not limited to theatre, speech-language, bilingual studies, gender and women’s studies, and linguistics.

## Theatre-based vocal training

Previous studies examining vocal training programs have focused on vocal techniques such as breathing control, vocal hygiene, and posture to improve vocal performance [13, 14]. In this study, the vocal techniques, language skills, and the self-perceived vocal empowerment of participants are examined. Focusing on these characteristics is important as young women need to have an awareness of their physical voice, as well as have the language to identify how they perceive their own voices to vocally empower themselves in public spaces. The SPEAK Vocal Empowerment Curriculum interweaves dramatic techniques and physical movement to enhance participants understanding of the biological, psychological, and emotional functions of voice production [4]. The Curriculum targets awareness of breathing, vocal relaxation, the development of resonance, and range, and offers participants the space to practice using their voices to address realistic community concerns their care about. When the Curriculum was implemented in Guatemala, Osnes and colleagues noted that participants’ self-evaluation improved after participating in the program. In their qualitative analyses, they also noted that participants were louder, spoke with more clarity, and had a reduced number of pauses, interrupted speech, and stammering. Importantly, they had a greater belief that their voice is important and may effectively contribute to their local and public communities [1]. In this study the first author adapted the SPEAK Curriculum for Egyptian participants. We were interested to see if the program would achieve similar results with participants from different cultural backgrounds, who spoke different languages. In another study, Abd El Hamid [15] examined the effectiveness of theatre in an Arabic classroom setting on the language skills of 30 fifth grade students in Egypt. The study tested reading out loud, silent reading, writing, students’ books, and teacher’s handbook. Abd El Hamid concluded that theatre is an effective tool to advance participants’ listening, speaking, and reading in Arabic, as well as their skills in other subjects, and increased their confidence and ability to articulate their thoughts and opinions [15]. She recommends the implementation of theatre curricula and teaching tools at schools, particularly Arabic classes, to advance language skills.

Unlike the vocal training programs that target speech performance, such as increased fundamental frequency and intensity [13, 14], the current study explored the impact of applied theatre on fundamental frequency. Previous research used these two measures to examine how listeners judge the speaker’s social characteristics [3, 16–18]. Fundamental frequency (*F0*) is a measure indicating the rate of vocal fold vibration [3, 19]. Fundamental frequency is associated with perceived vocal pitch. Based on a normative study [20], the average *F0* of English-speaking monolingual young women (251Hz) is significantly higher than that of young men (128Hz). A growing body of research suggests that voice pitch influences how speakers are perceived. For women, lower-pitched voices are perceived as more dominant and physically stronger [16, 17]. Research suggests their *F0* depends on a combination of external factors (e.g., the environment, barriers) and internal factors (e.g., self-perception; emotion) [3, 18, 21]. For example, Mattei and colleagues [18] examined the vocal characteristics of 41 women (age range: 18 and 52 years) across different conditions: control, physical distance, stress-competition, and both physical and stress conditions. They found that their participants’ *F0* was higher in the condition when both constraints were present. In addition, many studies have found that *F0* varies across language and language experience [22–25]. For example, a recent investigation found that Spanish-English bilingual speakers had higher *F0* compared with monolingual English speakers [22]. Another study found that Russian-English bilingual speakers who learned Russian (L1) at home had a consistently higher *F0* in Russian (L1) than in English (L2) during spontaneous speech [24].

For language performance, the current study examined the outcomes of lexical diversity, grammatical complexity, and speaking rates in both Arabic (L1) and English (L2). Although no previous studies have examined the effect of vocal training on language performance, there is a reason for hypothesizing that vocal empowerment training could interact with various internal and external factors and lead to the improvement of language skills. Increasing evidence indicates that there are interactions between language processing and speech motor performance [26, 27]. Additionally, the improvement of speech production could indirectly facilitate learners’ language skills. For example, in a series of studies, Kan and colleagues found that speech training facilitated monolingual and bilingual speakers’ initial word learning skills [28, 29]. Previous research has also demonstrated that increases in speech-motor coordination are associated with increases in utterance length and syntactic complexity [30]. In a study by Vonati who measured the MLU in Cypriot Greek-speaking Children, there is evidence that language sample analysis (LSA), of which the mean length of utterance (MLU) is foundational, legitimizes the ordinary talk of every child as a clinical resource and may be less vulnerable to dialect and cultural variations than traditional formal tests. It is considered an alternative language assessment procedure that brings cultural sensitivity, validity, accessibility and flexibility to the screening process [31]. In this study, the theatre-based vocal empowerment program provides opportunities for young women not only to learn about their physical voice but also to explore the links between their voice and societal issues through improvisation. The socio-cultural components in the vocal empowerment program and the interaction in group settings could lead to improved language skills [1, 32].

## Women’s voices in Egypt: Social characteristics

This study directly responds to formal national women’s empowerment initiatives and contributes to local feminist grassroot efforts. It presents young women an opportunity to practice using their voices for civic engagement by encouraging them to openly discuss and listen to each other’s perspectives about local, national, and international civic matters they care about [33]. Women have been at the forefront of national political movements in Egypt for centuries, though they are consistently underrepresented in leadership positions across fields, and in formal politics [34, 35]. Despite formal advocacy for women’s empowerment, wide gender disparities are present, including literacy rates, educational attainment, and labor force participation rates. In addition, unequal access to resources has led to women’s higher fear of failure and to their believing less in their capabilities, both of which have been shown to result in lower participation in the economic and political spheres [36].

National and local ministries within the Egyptian government acknowledge the need for systemic change to support women’s contributions. For example, the Egyptian Government’s *Sustainable Development Strategy*: *Egypt Vision 2030* [33], and the National Council for Women recommend increasing educational resources that widen young people’s understanding of civic engagement, and highlight the value of women’s contributions. Both propose that schools should facilitate discussions of civic responsibility, promote public speaking, school councils, and engage girls’ participation in matters that impact them [33]. Formal national efforts can only be successful if also supported in informal spaces, and studies indicate that young women in Egypt are “redrawing the boundaries of Egyptian national identity” [37]. Their feminist practice is characterized as decentralized and focused on artistic intervention [37]. Indeed, many young Egyptian women have recently increased their utilization of social media and visual art to cultivate new spaces to voice their opinions individually and collectively in the public sphere [38]. However there have been limited avenues for young women to gain awareness of their physical, psychological and social voice, in order to feel vocally empowered to express themselves in in-person settings as well as virtual. Hence the focus of this study.

When reflecting on young women’s vocal empowerment in Egypt, it is essential to consider their social-economic status, the types of schools they attend, and the languages that they speak. These differences are likely to influence participants’ choice of expression, and guide their experiences in their communities, furthering the need to investigate participants in contrasting Egyptian cities. We chose to implement this study in Alexandria and Aswan, two historical coastal cities, that exemplify the socio-economic and cultural differences and inequities that exist across governates, which can be distinguished by a rural-urban divide, and by the division between Lower and Upper Egypt. Alexandria, located in Lower Egypt, is the second most populous city in the country, after Cairo, and it is also one of the country’s most significant economic centers [38]. Its population is approximately 5.28 million. Alexandrians predominantly speak the dominant Cairene Arabic dialect, which is considered the most “prestigious” variety in the country. Aswan is in Sa’id Masr, a region in Upper Egypt that spans from southern Cairo to the Sudanese border. It has a population of two hundred thousand [39]. The Arabic dialect spoken there is distinct from Cairene Arabic phonologically, semantically and even morpho-syntactically [40]. It is associated with rural non-urbanized communities, and generally looked down upon [41]. The survival of, and the pride associated with speaking an Upper-Egyptian dialect amidst all the pressure from a highly centralized Egypt for all Egyptians to speak Cairene Arabic is noteworthy, particularly for this study on young women’s vocal empowerment [40].

Another important factor for this study is young women’s exposure to and use of English as a second language and their education experiences in each city. Egyptians have been exposed to multiple foreign languages, consequent to centuries of colonization and tourism. The British occupation from 1882–1956 made English the principal foreign language of the country [42]. Today, there is an increasing recognition that English is a powerful tool in society, to the point that a mere British or American accent can result in a pay raise [43]. Most bilinguals in Egypt are considered sequential language learners, as they are exposed to Arabic from birth then learn a second language—typically English—at school [42], however, the socio-economic status of a family is likely to determine the child’s proficiency in this new language, based on their access to education.

There are four types of schools in Egypt that may correlate to socio-economic differences; public, national institutions, private, and embassy schools [44]. The Egyptian Ministry of Education administers and funds public schools, which educate 92% of the population. Public schools are divided into two main types: schools where the Egyptian curriculum is taught in Arabic, and experimental schools that teach mathematics and science in English or French, with the remainder of the subjects being taught in Arabic [44]. Some national institutions are public, others are private and are funded by social or national religious endowments [44]. Private schools, which have grown in popularity, offer a combination of the national curriculum and international diplomas, while others like embassy school, adhere to a strictly international diploma system [44].

Rural governates, like Aswan don’t have any private schools with international diplomas or embassy schools. Parents in these governorates, or members of low-income families therefore have less exposure to international education and are more likely to be Arabic monolinguals, limiting their ability to develop their language skills outside of school. Whereas students who attend private international schools generally come from a higher socio-economic backgrounds and advanced bilingual households, where English may also be spoken at home. They have greater access to educational resources, international travel, and media that immerses them in their second language [44]. This exposure heightens their appreciation of English, as they recognize it as a global language of communication, a presumed sign of intellect and superior education, as well as a status symbol in Egypt, ultimately resulting in the shift from (L1) to (L2) language dominance. Participants self-perceptions of their language use is noteworthy in this study.

## The current study

The purpose of this study was to examine the effect of a theatre-based vocal empowerment program on young Egyptian women’s vocal and language characteristics. To gain a preliminarily understanding of the outcomes of the theatre-based vocal empowerment program, we employed a quasi-experimental design, with all participants receiving the theatre-based vocal empowerment program.

Different from previous studies on vocal training, this study examined not only vocal outcomes but also language characteristics because applied theatre fosters a supportive community space that encourages participants to improvise and think creatively to visualize how they may react to realistic situations that they may encounter in their daily lives. In speech production, we examined fundamental frequency (*F0*). In the area of language production, we focused on the lexical characteristics, grammatical complexity, and speaking rate (i.e., words per minute in Arabic (L1) and in English (L2). In addition, we examined whether participants’ self-perception of confidence in their voices changed after the theatre-based vocal empowerment program. We examined whether participants from Alexandria and Aswan, with different socio-economic and linguistic backgrounds, respond to the theatre-based vocal empowerment program differently. Results can potentially inform the implementation of future studies that meet the specific needs of the communities. The following specific research questions are addressed:

1. Is there any effect of the theatre-based vocal empowerment program on young women’s vocal and language characteristics in Egypt?
2. Do participants from Alexandria and Aswan respond differently to the theatre-based vocal empowerment program?
3. Is there a significant difference between the outcomes in Arabic (L1) and English (L2)?
4. How do young women perceive their voice before and after the theatre-based vocal empowerment program? Are there any differences between young women from Alexandria and Aswan?

## Methods

### Participants

Prior to the study, an Institutional Review Board (IRB) approval was obtained from the University of Colorado Boulder. The theatre-based vocal program was free of charge and open to local Egyptian girls from private or public schools. Fifty-five native Egyptian participants in Alexandria (n = 19; mean age = 13.5) and Aswan (n = 36; mean age = 14.05) were recruited for this study. All participants were bilingual, Arabic (L1) and English (L2). The Cambridge English Testing Centre at the Arab Academy for Science, Technology, and Maritime Transport (AASTMT) recruited the participants by distributing informational flyers to parents interested in the Centre’s summer school programs.

In Alexandria, all the participants attended a private school. Ten attended private school with a national curriculum, one attended an embassy school, and eight attended an international curriculum, indicating a higher socio-economic status than participants in Aswan. In Aswan, twenty-one participants attended public school, ten attended an experimental school, and only five attended a private school with a national curriculum. Participants in Alexandria indicated a preference of speaking and responding to the program activities in English over Arabic, while in Aswan, there was a preference for Arabic. All participants were sequential (L2) learners, who learn Arabic as a home language (L1) and English as a second language (L2). Consequent to the type of schools participants attended in the different cities, participants in Alexandria were early sequential and in Aswan they were late sequential. None of the participants had a theatre background or exposure to the exercises before the study. All participants attended the full 12 consecutive sessions.

### Collection of data

The first author met with two groups of participants in Aswan and one in Alexandria on the AASTMT campuses. AASTMT has numerous branches around the Middle East, and the Centre is the sole entity on campus with a dedicated summer school for youth. The entire program lasted for 12 consecutive days, including 2 sessions for the pre- and post- measures, 10 90-minute sessions over 10 days, and a public sharing session between the 11th and 12th days. Each participant’s vocal and linguistic characteristics, and self-perception of their voice was tested before and after the theatre-based vocal empowerment program. The assessment materials were adapted from the SPEAK curriculum [5]. The self-assessment questionnaire was written in both Arabic and English, with the Arabic statements on the right-hand side and their corresponding English statements on the left. Participants were given the single sheet questionnaire and asked to respond to the questions. They had the option to respond in either language. This was intentional as a further method of assessing their language preference. An iPad was the only piece of technology used for data collection. There was insufficient time to allow for clearance of other devices through Egyptian customs, given the duration of travel and time limitations for conducting the study. Participants were recorded responding to two speech production tasks: (1) spontaneous speech and (2) reading aloud. They were recorded using the Voice Analyst app on an iPad. It was placed vertically facing the participants, 30 cm away from their mouths. Each participant was recorded separately, and none of them were given the prompts or the reading passages in advance. All the recordings took place in the same classroom in each city. The first author delivered the instructions before participants started reading the passages and completing the vocal self-perception assessment survey and did not offer assistance during the assessment.

### Outcome measures

Three measures were used to examine participants’ perception of their voice and their speech production characteristics before and after the theatre-based vocal empowerment program. The outcome measures were initially developed by Osnes and Hackett [1], and were adapted to Arabic and English by the first author [45].

#### Speech production tasks

To elicit participants’ connected speech, we implemented two speech production tasks in this study: (1) spontaneous speech and (2) reading aloud. In the spontaneous speech task, all participants were asked to respond to the following prompts in both Arabic and English: 1. Hello my name is…, 2. My voice is important to me because…, 3. My greatest community concern is, 4. One idea I have to improve my community is to…. The recording started as soon as the participant started reading the first prompt. Participants were not required to respond to the questions within a set time frame. They could take as much time as they needed to respond to each of the prompts. The recording stopped after the participant was finished responding to all four prompts.

For the reading aloud task, participants were instructed to read aloud two different passages, one in Arabic (see S2 Appendix), and one in English selected from a third-grade reading book used at public schools in Egypt (see S3 Appendix). There were 56 words in the Arabic reading passage and 41 words in the English reading passage. Each task completed first in Arabic, followed by English. Participants were not given the passage to read before recording. They were recorded the first time they saw the passage.

#### Vocal self-perception survey

The Vocal Self-Perception Survey, adapted from the SPEAK curriculum, was used to examine participants’ self-perception of their voice. There was a total of 13 questions (see S1 Appendix). The first 12 questions required participants to respond by ticking boxes. The original SPEAK material had the categories: ’Always True,’ ’Mostly True,’ ’Sometimes True,’ ’Never True.’ In this study, we added a ’I Don’t Know’ option. The thirteenth question was measured on a scale ranging from 1–10, with 1 associated with feeling the most positive, and 10 being the most negative, indicated by drawings of smiling and frowning face and number. Three separate charts were used for question thirteen to assess participants’ feelings about their voices at home, school, and public. After completing the assessment individually, participants marked their preference in the group assessment sheet on the wall to compare their feelings. These surveys were written with English on the left-hand side and Arabic on the right. No instruction was given about which language to complete the assessment material to evaluate participants’ language preference. There were significant differences in language preference across the two groups of participants (*β* = 4.97, *SE* = 1.16, *χ*^2^ = 18.48, *p* < .001). Thirty-two participants (97%) from Aswan completed the form in Arabic, while only 1 participant (5%) from Alexandria completed the form in Arabic. Participants used the same language in the pre- and post-assessment. The analysis was done based on their responses in each language.

### Theatre-based vocal empowerment program

Two groups in Aswan and one in Alexandria met for 90-minute sessions over 10 consecutive days in each city. The sessions were conducted in Arabic in both cities. Participants in Alexandria responded in both Arabic and English and sometimes asked clarifying questions in English. Participants engaged in daily vocal warm-up, and applied theatre games adapted from Linklater [4] and Boal [46]. The exercises, most of which were repeated daily, engaged participants in various approaches to support their understanding of their physical voice to increase their self-authorship and belief in their voices to use in public. Each session started with a relaxation visualization to set the intention for the day, followed by a group building and vocal empowerment program. Some of the exercises were modified to adapt to bilingual Arabic-English participants in an Egyptian context. For example: adding Arabic letters, with sounds that do not exist in English (ض، ث، ق، ف) to the daily vocal warm-ups. The daily exercises emphasized a supported voice, encouraging participants to reflect on the multiple aspects of voice production. The day concluded with a critical reflection on the purpose of the exercises and offered participants a space to voice their opinions, and finally closing.

The first six sessions focused on understanding the mechanics of the physical voice, whereas the final six focused on participants taking their voices in public. The pre-program assessments were taken during first session. In the second session, participants identified the values of their communities and the characteristics of an empowered voice. The third session focused on the daily vocal empowerment program to expand the vocal range, improve posture and practice speaking clearly in a supported and healthy way. These daily exercises were repeated at the beginning of every session thereafter. These included producing the letters AEIOU while stretching, rolling up the spine, whispering "shh," singing "ahh," and repeating an every-day phrase, with arms extended at a high, middle, and low level at each time. In the fourth session, participants started devising a vocal affirmation song to represent their empowered voice. The original SPEAK curriculum outlined a specific song; however, in Egypt, participants in each city chose to author original vocal empowerment songs that alternated between Arabic and English verses. In the fifth session, participants learned about the components of their physical voice, understanding the power (lungs, diaphragm), source (vocal cords), and filter (jaw, teeth, tongue, lips). The activities were done through a specific program to facilitate the participants to notice the differences between each of these areas individually, anatomic diagrams, and watching a laryngoscopy video and physically embodying these mechanisms through acting out the different functions to understand how they operate entirely.

The sixth session emphasized expressing and listening to others’ opinions. It focused on community building, allowing participants to voice their goals for their voices, their career and community hopes for the future, and how they will use their voice to support others. The seventh session encouraged participants to practice using their voice in public. In Aswan, the participants went to the Aswan Botanical Garden. In Alexandria, they went to the university courtyard. In the eighth session, participants created their declarations by writing down the responses to the spontaneous speech recording prompts. The ninth and tenth sessions intended to cultivate community. Participants generated short skits to portray the current state of the community issue, the desired future solution, and the transition of how to get from the current state to the future one. The exercises facilitated conversations surrounding the following issues: an understanding of what vocal empowerment is, how participants perceive it, and what participants’ community concerns are on a local, national, and international level. Participants reported feeling more comfortable and willing to be louder and clearer every day.

In addition, participants completed a daily journal after every session. The journal had writing prompts in both Arabic and English. Participants were free to respond in either language. In Aswan, participants generally wrote in Arabic or Franco-Arabic, whereas most participants in Alexandria wrote in English or Franco-Arabic. The daily journals were analyzed using qualitative measures and reported in another study by the first author [45]. After the final session, participants presented their skits, song, personal declarations, and ideas about vocal empowerment in a celebration attended by their friends, families, and teachers. Participants were given the option to present in English or Arabic. In Aswan, the President of AASTMT and the Governor of Aswan also attended the celebration.

### Data coding and analysis

The vocal self-perception survey administered pre- and post- the implementation of the vocal empowerment program was coded as (1) “Never True,” (2) “Sometimes True,” (3) “Mostly True,” (4) “Always True.” The “I Don’t Know” category was analyzed separately. Nonparametric tests were used to compare the two groups of participants (Aswan vs. Alexandria) and their pre- and post-intervention performance.

The audio recordings of the two speech production tasks—spontaneous speech and reading aloud—in Arabic and English were analyzed individually using Praat, a software for analysis of speech in phonetics [47] command line to extract the *F0* contour. Unlike the reading aloud task in which all participants read the same passage in (L1) and (L2), the spontaneous speech varied across participants. Therefore, participants’ Arabic and English spontaneous speech was transcribed and analyzed. The language samples were analyzed using *Systematic Analysis of Language transcripts* (SALT), a software for analyzing language samples [48]. Multivariate analysis of variance (MANOVA) was conducted to determine the extent to whether participants’ social cultural-language backgrounds affect their speech outcomes after the theatre-based vocal empowerment program. The vocal characteristic was fundamental frequency (*F0*). The language measures include the mean length of utterance in words (MLU-w), number of different words (NDW), words per minute (WPM). The independent variables were group (Aswan vs. Alexandria), language (Arabic vs. English), and theatre-based vocal empowerment program (pre vs. post). This study examined MLU-w since it is more reliable, and less arbitrary than MLU-m. It is considered a neutral procedure that can be used reliably across various languages and dialects, it maintains cross-linguistic consistency and comparability and can be applied successfully in bilingual and dialectal research [31].

## Results

### Spontaneous speech: Vocal and language outcomes

**Fig 1:**
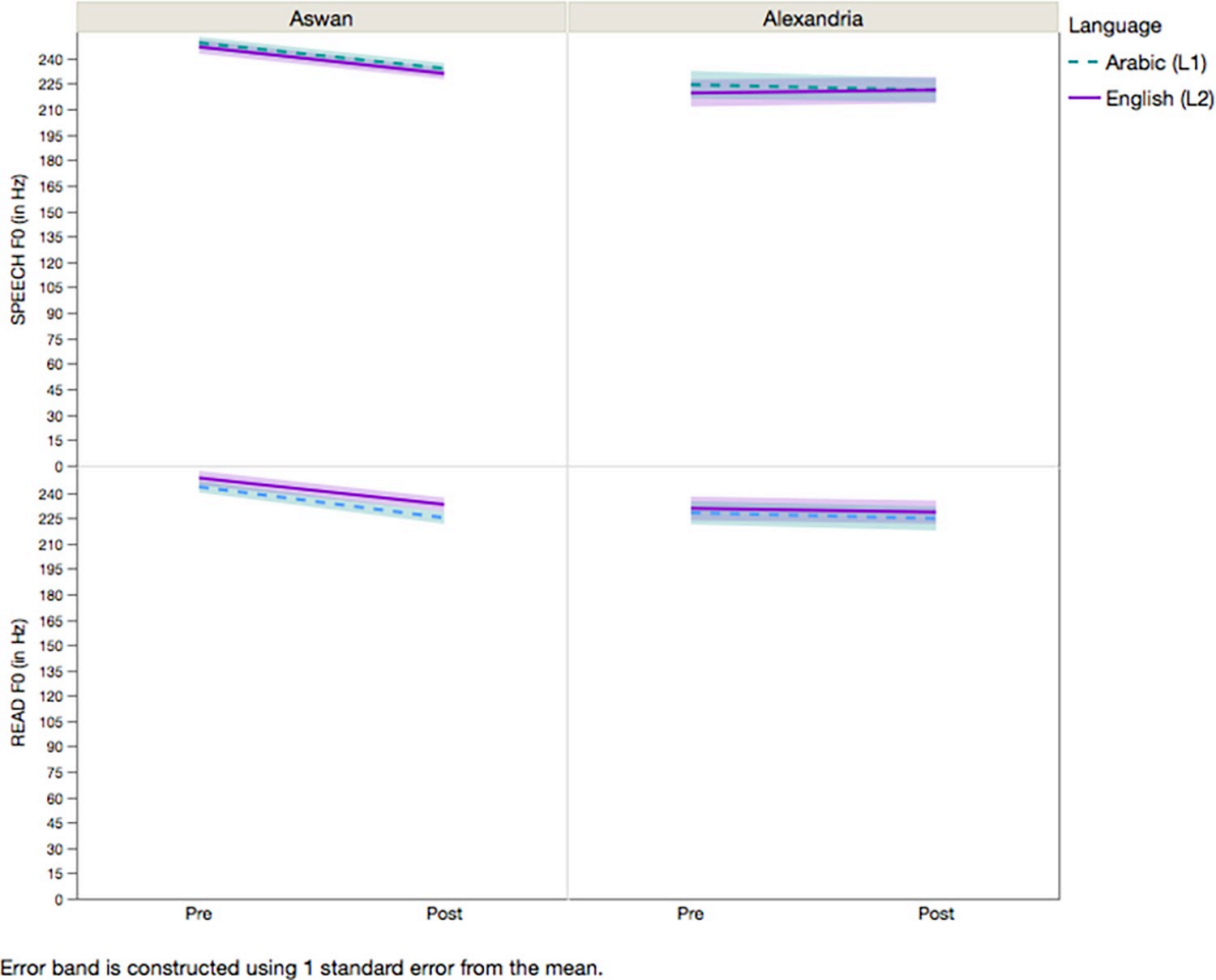
Means and standard deviations (error bands) of the fundamental frequency (*F0*; in Hz) across the spontaneous speech tasks and reading tasks in Arabic and English for Aswan (left) and Alexandria (right) group before and after the theatre-based vocal empowerment program.

Participants’ vocal and language characteristics of the spontaneous speech in Arabic and English before and after the theatre-based vocal empowerment program are summarized in Table 1. *F0* was examined in the spontaneous speech of the participants. The overall MANOVA results are summarized in Table 2. Results showed a main effect of the theatre-based vocal empowerment program on *F0*. After the theatre-based vocal empowerment program, the results suggest that participants had higher *F0*. There was also a main effect of group on *F0*. Participants from Aswan had lower *F0* than those from Alexandria. However, there is no significant main effects of language on any speech variables, suggesting participants’ speech characteristics were similar across Arabic and English. Results showed that there was a significant interaction between group and theatre-based vocal empowerment program for *F0*. For the Alexandria group, their *F0* remained the same after the program. For the Aswan group, their *F0* after the program was significantly lower than their F0 before the program (see Fig 1).

**Table 1:** Means and standard deviations (in parentheses) of the language characteristics of the spontaneous speech of the participants from Aswan and Alexandria.

Aswan (n = 36)				
Parameter	Arabic (L1)		English	
	Pre	Post	Pre	Post
Fundamental Frequency (F0)	248.94 (22.21)	233.79 (19.90)	246.56 (23.91)	230.80 (19.76)
Mean length of utterance (MLU)	9.44 (4.05)	9.45 (2.77)	9.5 (3.7)	12.1 (5.1)
Number of Total words (NTW)	38.33 (16.18)	38.36 (13.62)	36.5 (20.5)	48.1 (20.9)
Number of Different words (NDW)	32.39 (9.86)	33.92 (9.28)	30.6 (12.1)	37.9 (13.3)
Word Per Minute (WPM)	94.19 (19.98)	123.66 (14.88)	80.8 (4.1)	100.6 (21.5)
Alexandria (n = 19)				
Parameter	Arabic (L1)		English	
	Pre	Post	Pre	Post
Fundamental Frequency (F0)	224.27 (35.52)	221.12 (30.60)	219.38 (34.76)	221.16 (33.68)
Mean length of utterance (MLU)	7.78 (2.91)	7.65 (3.04)	10.3	14.5 (4.5)
Number of Total words (NTW)	21.20 (14.9)	22.56 (18.68)	35.3	58.7 (21.7)
Number of Different words (NDW)	15.23 (8.98)	16.17 (11.76)	24.4	39.4 (13.7)
Word Per Minute (WPM)	83.88 (23.2)	108.77 (16.78)	110.27 (23.5)	156.21 (27.22)

**Table 2:** Summary of the MANOVA results of the fundamental frequency (F0) for the spontaneous speech and reading aloud tasks.

	*F0*			
	Spontaneous speech		Reading	
	*F (1*, *52)*	η2	*F (1*, *52)*	η2
Language	3.26	.06	1.29	.00
Group	7.89*	.13	35.46***	.41
Vocal empowerment program	7.36*	.12	1.60	.03
Language x Group	0.02	.00	0.61	.00
Vocal empowerment program x Group	6.13*	.11	5.76*	.10
Language x Vocal empowerment program	0.69	.01	0.22	.00
Language x Vocal empowerment program x Group	1.22	.02	1.71	.03

*p < .05 **p < .01

***p < .001.

**Fig 2:**
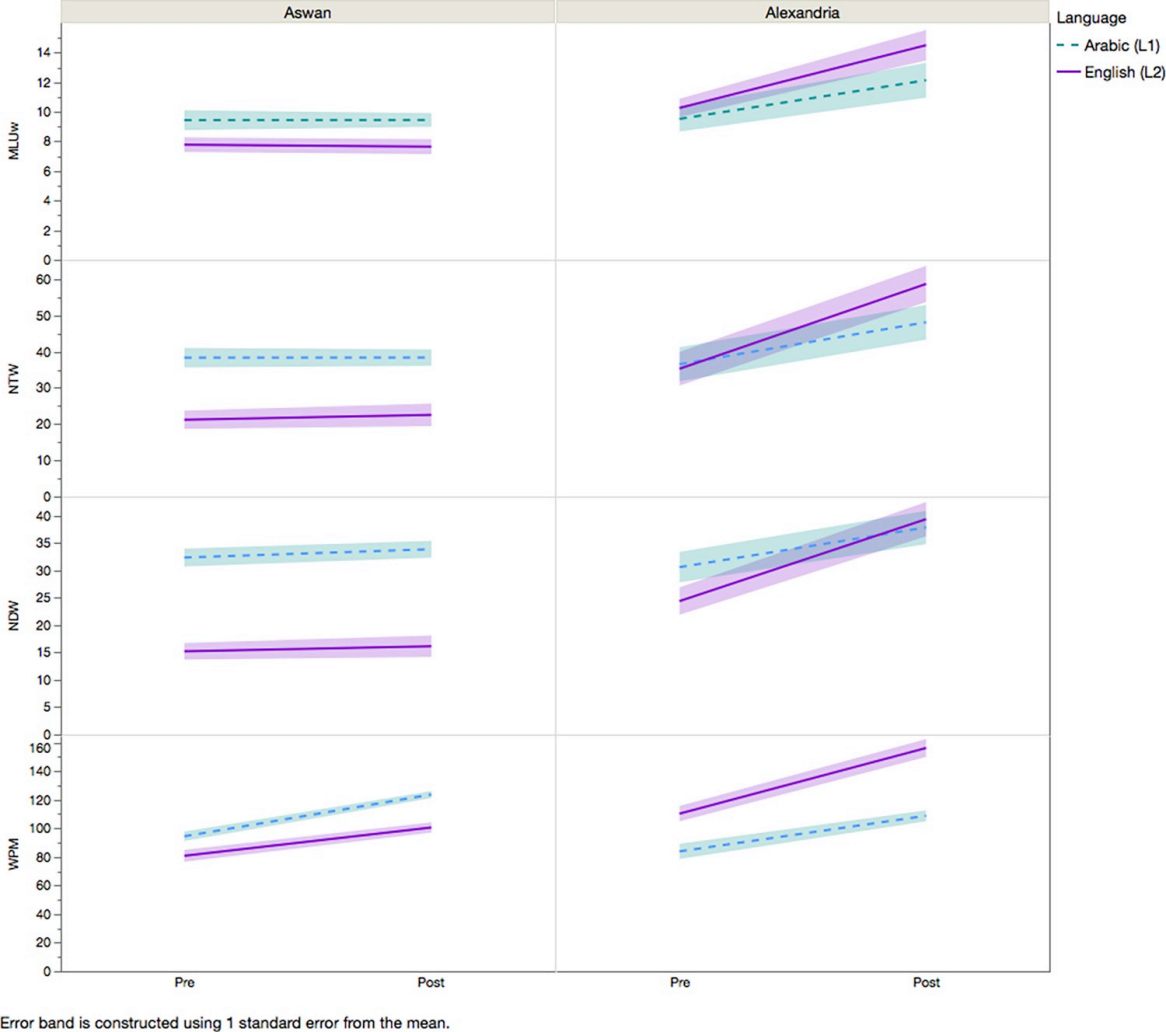
Means and standard deviations (error bands) of the mean length of utterance (MLU), number of total words (NTW), number of different words (NDW), Words per minute (WPM) of the spontaneous speech tasks in Arabic and English for Aswan (left) and Alexandria (right) group before and after the theatre-based vocal empowerment program.

Four language measures, including the mean length of utterances in words (MLU-w), number of total words (NTW), number of different words (NDW), and words per minute (WPM,) were examined in the spontaneous speech of the participants. MANOVA results (see Table 3) showed that there were main effects of the theatre-based vocal empowerment program on all language measures, including MLU-w, NTW, an, NDW, & WPM. The results suggest that participants had longer MLU-w, more total words, greater lexical diversity (indicated by NDW), and more words per minute in the spontaneous speech after the program. However, the results of MLU-w, NTW, and NDW should be interpreted together with the significant interactions between group and theatre-based vocal empowerment program. The Alexandria group had significantly greater gains if MLU-w, NTW, and NDW after the program than the Aswan group (see Table 3 and Fig 2). MANOVA results also showed that there was an effect of language on NDW, NTW, & WPM. The findings suggest that participants produced more total words (NTW) and used more diverse vocabulary (NDW) in Arabic than in English but had greater rate of speech (WPM) in English than in Arabic. However, the results of the significant interaction between language and group indicated that the Aswan group had overall longer MLU-w, more total words (NTW), more diverse vocabulary (NDW), and higher speaking rate (WPM) in Arabic than in English. In contrast, the Alexandria group had longer MLU-w, more total words (NTW), more diverse vocabulary (NDW), and higher speaking rate (WPM) in English than in Arabic.

**Table 3:** Summary of the MANOVA results of the language measures for the spontaneous speech task.

	MLU-W		Number of Different Words		Number of Total Words		Words Per Minute	
	*F (1*, *52)*	η2	*F (1*, *52)*	η2	*F (1*, *52)*	η2	*F (1*, *52)*	η2
Language	.05	.00	51.54***	.5	7.4**	.12	51.54***	.5
Group	22.59***	.26	16.15***	.23	16.46***	.24	16.15***	.23
Vocal empowerment program	10.55**	.17	16.26***	.24	13.69**	.21	16.26***	.24
Language x Group	14.87***	.20	30.09***	.37	23.49***	.31	30.09***	.37
Vocal empowerment program x Group	11.35**	.07	10.54**	.17	11.71**	.18	10.54**	.17
Language x Vocal empowerment program	1.28	.02	3.25	.06	4.46*	.08	3.25	.06
Language x Vocal empowerment program x Group	1.33	.00	4	.07	2.54	.05	4	.07

*p < .05

**p < .01

***p < .001.

### Reading aloud: Speech outcomes

Participants’ speech characteristics of the reading aloud task in Arabic and English before and after the theatre-based vocal empowerment program are summarized in Table 1. *F0* was examined in the reading aloud task. The overall MANOVA results are summarized in Table 2. Results showed that there was no effect of theatre-based vocal empower program on *F0*, suggesting that participants, collapsed across the two groups, had similar *F0* after the theatre-based vocal empowerment program. There was also a main effect of group on *F0*. Participants from Aswan had lower *F0* than those from Alexandria. Results also showed that there was a significant interaction between group and theatre-based vocal empowerment program for *F0*. For the Alexandria group, their *F0* remained the same after the program. In contrast, the *F0* of the Aswan group after the program was significantly lower than their *F0* before the program (see Fig 1).

### Vocal self-perception survey

Wilcoxon Signed Ranks Tests were used to examine the questionnaires. Results showed no significant differences between the Alexandria and Aswan groups. However, within each group, participants had improved vocal self-perception after the theatre-based vocal empowerment program (see Table 4). Both groups had higher ratings after the theatre-based exercises about the role of their body in voice production (Questions 2), about feeling good and not ashamed to speak (Questions 4 & 7,) and being heard and understood at home and school (13a and 13b). However, there are differences between the two groups. The Aswan group’s ratings significantly increased after the program for question 3 (*I understand what parts of my body produce my voice*) and 5, (*I feel my voice is mine and belongs to me*). However, the Alexandria group did not significantly change ratings for those questions after the program. The Alexandria group’s ratings significantly increased after the program for question #10 (*I use my voice to share my feelings and ideas*.). However, the Aswan group did not have any change of ratings for that question after the program.

**Table 4:** Summary of the vocal self perception survey results from Aswan and Alexandria.

Items	Aswan (n = 36)	Alexandria (n = 19)
	*Z*	*Z*
1. People can easily hear and understand my voice.	1.56	2.33*
2. I use various parts of my body when I express myself. (Example: I gesture with my hands)	3.62***	2.64*
3. I understand what parts of my body produce my voice.	3.11***	1.40
4. I feel good about my voice.	2.17*	2.72*
5. I feel my voice is mine and belongs to me.	2.67*	1.41
6. I feel safe using my voice to share my feelings and ideas.	1.90	1.51
7. I am not ashamed to speak.	2.65*	2.22*
8. I feel my voice has the power to make a positive impact for my life and others.	0.84	1.58
9. I listen to and consider what other people say.	1.49	1.47
10. I use my voice to share my feelings and ideas.	1.35	2.89*
11. I use my voice to help myself and others.	2.00	0.30
12. I use my voice with responsibility and courage.	2.00	1.59
13a. I am heard and understood at my home	2.15*	2.07*
13b. I am heard and understood at my school	2.30*	2.35*
13c. I am heard and understood in public	1.96	1.89

*Note*.

*p < .*05* **p < .*01*

***p < .*001*.

The *p* values of the *Z value*s underlined were .05.

## Discussion

The overall goal of this study was to examine the effect of a theatre-based vocal empowerment program on vocal and language skills and self-perception of vocal empowerment of young women from Alexandria and Aswan, in Egypt. The theatre-based young women’s vocal empowerment program in this study offers one method to address the needs expressed by the OCED and the National Council for Women to invest in educational resources that encourage and widen young people’s understanding of civic engagement, promoting public speaking, and girls’ participation in matters that impact them. Theatre is a community-based, collective activity that engages young women in an embodied way to practice using their voices with confidence to speak up about community concerns they care about. Applied theatre empowers them to believe in themselves from a young age and encourages them to continue pursuing civic action later in life.

This study is the first to investigate the contribution of socio-cultural diversity factors to the outcomes of a theatre-based vocal empowerment program. Young women from Alexandria and Aswan were examined. Several participants in both cities indicated that this program was the first experience they were taken seriously and could vocalize issues they cared about. These young women had different preferences of the language spoken and attended different schools. Though all the young women are sequential L2 learners, they came from different socio-economic and educational backgrounds, which may have impacted the outcomes. In Alexandria, all the participants attended a private school, and they indicated a preference for English (L2) over Arabic (L1). Whereas, in Aswan, most participants attended a public school and showed a preference for Arabic (L1). The results of this study showed that participation in applied theatre exercises has an impact on participants’ vocal and language production skills and their vocal empowerment knowledge. However, this study does not include a control group, and we could not rule out whether the effects were due to other factors such as the testing effect. Despite this limitation, there are two significant findings in this study. First, there were differences between the two groups of women (Alexandria vs. Aswan) in their vocal and language outcomes after the theatre-based vocal empowerment program. The second important contribution is that, although both groups made significant gains in some self-perception survey questions there were differences between the women from Alexandria and Aswan regarding the gains for certain questions. Outcomes of this study suggest that participants’ socio-economic, language acquisition, and geographical backgrounds are essential factors that impact both the implementation and the outcomes of a theatre-based vocal empowerment program.

### Fundamental frequency (*F0*)

In this study, participants had higher *F0* during spontaneous speech after the theatre-based vocal empowerment program. The result is compatible with results from the SPEAK curriculum conducted in Guatemala [1]. One important contribution of this study is the finding indicating the differences between the two groups of women from different backgrounds. The results of both spontaneous speech and reading tasks indicated that the *F0* of the young women from Aswan declined after the program, whereas the *F0* of the participants from Alexandria remained the same after the program. Previous studies showed that lower-pitched voices in women are perceived as more dominant and stronger [16, 17]. This is linked to the language of the study. In some cultures, lower-pitched voices may not be perceived as more dominant. However, it is applicable to the Arabic and English languages in an Egyptian context. This study did not explicitly teach the participants the link between perceived societal impressions of lower-pitched voices and personality. Although the data in this study are not sufficient to link the *F0* decline in the Aswan group and their self-perception of themselves, the *F0* decline raises an interesting question about whether and how the theatre-based vocal empowerment program indirectly affects women from a rural community in Egypt. The city of Aswan is significantly smaller in size than Alexandria, and people from Aswan, on average, have a lower SES. The city of Aswan is more community-driven, with a stronger desire for collaborative community ties in comparison to the more individualistic society of Alexandria. Several of the participants in the program knew each other from school. By the end of the program, all the young women in the Aswan group exchanged phone numbers and social media contact information. They created a WhatsApp group chat with the first author that has remained active ever since. The young women have since arranged meetups and study sessions together. When they found out the first author was visiting Aswan the following year, they regrouped to meet informally. This community experience was not replicated in Alexandria. The participants from the Alexandria group did not appear interested in remaining in contact. These differing approaches post-program may be linked to the city atmosphere and feelings of belonging. Future research should fine-tune the possible effects of socio-economic and language factors on the learning outcomes.

### Lexical and grammatical outcomes

In the current study, the theatre-based vocal empowerment program engages participants in various embodied exercises that allow participants to think about and vocalize their thoughts and feelings about various community concerns. Numerous participants in both cities indicated that this was the first time they had space to safely express their thoughts about issues such as education, sustainable development, and socio-economic disparities [45].

These exercises motivate young women to speak more freely with confidence in their ideas and not hold back. Therefore, assessing lexical outcomes is essential. Unlike other vocal training programs that focus on vocal performance, the current study is the first to examine young women’s language outcomes. An important finding in this study is the differences between the group of women in their lexical-grammatical outcomes. The young women from Alexandria had significant grammatical complexity gains, the number of words produced, lexical diversity, more words per minute after the program, compared to the Aswan group (see Table 3). It is possible that the Alexandria group developed greater skills to state their opinions in a more succinct manner. Another interesting finding is the interaction between language and group. The Aswan group produced longer sentences, more words, more diverse vocabulary, and a greater speaking rate in Arabic than in English. In contrast, the Alexandria group had produced longer sentences, more words, more diverse vocabulary, and a greater speaking rate in English than in Arabic. Their English skills and preferences may be due to their increased English exposure though school, social media, and social settings, given that Alexandria is more globalized than Aswan. Taken together, the findings suggest that the theatre-based vocal empowerment programs appear to facilitate young women’s stronger language.

### Self-perception vocal empowerment knowledge

The theatre-based vocal empowerment program in this study targets the technical components of the voice and the emotional and psychological aspects. Participants’ self-perception assessments pre- and post-program are essential factors in working towards vocal empowerment. This signifies how they feel about their voices and how they may use it in the future. As indicated by the lexical and grammatical and voice outcomes, participants responded differently to the self-perception questions, based on the city. Participants showed a significant difference in responses to questions: "2: I use various parts of my body when I express myself (example: I gesture with my hands)". "4: I feel good about my voice". "7: I am not ashamed to speak". "13a: I am heard and understood at my home". "13b: I am heard and understood at my school". These results present a positive impact on participation in the program. One of the main goals of this program was to increase young women’s belief in themselves, their comfort in their bodies, and to reduce feelings of shame surrounding using their voice. Though participants have varying socio-economic and cultural backgrounds, they are all impacted by the patriarchal constructs of gender, that influence their voices. In this program, participants were encouraged to speak up freely and express their thoughts, even if they challenged or contradicted other group members and the facilitator’s ideas. They participated in various exercises that focused on posture, breathing technique, and created scenarios of addressing community issues they cared about with family members and their friends.

The participants responded differently to some of the questions. One possible explanation is that participants’ language preferences may contribute to the results. The Alexandria group showed a preference for responding in English, whereas the Aswan group wrote in Arabic. The Alexandria group showed significant improvement in questions "1: People can easily hear and understand my voice" and "10: I use my voice to share my feelings and ideas". In contrast, the Aswan group showed significant improvement in questions "5: I feel my voice is mine and belongs to me, and "3: I understand what parts of my body produce my voice". It is unclear to what extent cultural and socio-economic factors affect participants’ self-perception. More studies are needed to investigate the differences thoroughly. These findings raise questions about the development of questions for self-assessment for future studies.

## Limitations

There are several methodological limitations of the present study. One of the main caveats is that the current study does not include a control group. We cannot determine whether the vocal and language outcomes were due to the theatre-based vocal empowerment program or other factors such as the testing effect. Another limitation was related to how the participants were sampled and how we obtained information about their diverse backgrounds. Participants recruited from Alexandria and Aswan were a convenience sample. The sample might not be representative of the diverse young women in Egypt. At the time of testing, our participants spoke Arabic as a home language (L1) and learned English as a second language (L2). We did not directly measure their language ability or use in Arabic and English or obtain information such as their exposure to Western media, their cultural attitudes, and their desire to publicly use their voice. Future studies should adjust assessment factors, including a control group and control for bilinguals’ socio-economic, cultural, and language experience factors. Additionally, participants’ spontaneous speech and reading were recorded in a quiet room using the Voice Analyst app on an iPad due to the limitations of the allotted time for customs clearance of technological devices. Many acoustic factors, such as background noise and reverberation time, were not taken into consideration. Lastly, although the results indicate positive outcomes, the long-term effects remain unknown. Researchers should cultivate a plan for long-term follow-up, assess the program’s impact, and provide continued support.

## Conclusion

The current study shows promising results for the implementation of theatre-based vocal empowerment programs in the community. Although it is common to believe that the patriarchal system limits women’s belief in their self-authorship and controls their societal expectations, young Egyptian women are not homogenous. One crucial finding is the differences in the vocal and language outcomes between participants from Alexandria and those from Aswan. Results suggest that socio-economic, educational, and language backgrounds are essential factors affecting learners’ outcomes in vocal empowerment programs. Although this study was conducted in Egypt, these results can be informative for other community outreach programs. When designing international outreach programs, especially vocal empowerment programs, researchers must consider participants’ socio-economic, cultural, educational, and language diversity in the communities. It is necessary for organizations working towards international women’s rights and empowerment, including those implementing theatre-based or artistic programs, to be conscious of language proficiency, socio-economic status, culture, and education levels in communities they will work with.

## Supporting information

S1 Appendix (PDF)

S2 Appendix (PDF)

S3 Appendix (PDF)
