# Supplementary material for: The effects of theatre-based vocal empowerment on young Egyptian women’s vocal and language characteristics
Source: PLoS One. 2021 Dec 31;16(12):e0261294. doi: 10.1371/journal.pone.0261294 (PMC8719750; doi:10.1371/journal.pone.0261294)
Supplement: S1 Appendix — (PDF) [file pone.0261294.s001.pdf]

Vocal Efficacy Scale – مقياس الكفاءة الصوتية

Name / الاسم:____________________________ Date / التاريخ :_________________

For each statement, mark with an X whether the statement is Never True, Sometimes True, Mostly True or Always True in the boxes below.

ضع علامة x امام كل عباره من العبارات التالية

	Never True	Sometimes True	Do not know	Mostly True	Always True	حقيقي دائما	حقيقي معظم الوقت	لا أعرف	احيانا حقيقي	غير حقيقي على الاطلاق	
EXAMPLE: 0. I can use my voice to speak.				X							مثال: انا استعمل صوتي لكي أتكلم.
1. People can easily hear and understand my voice.											١. الناس يستطيعون سماع وفهم صوتي بسهولة.
2. I use various parts of my body when I express myself. (example: I gesture with my hands)											٢. انا استعمل أجزاء عديده من جسمي للتعبير عن نفسي. (مثال: استعمل يدي للتوضيح).
3. I understand what parts of my body produce my voice.											٣. انا افهم كيف جسدي ينتج صوتي.
4. I feel good about my voice.											٤. انا اشعر بالرضي تجاه صوتي.
5. I feel my voice is mine and belongs to me.											٥. انا اشعر ان صوتي ملكي ويخصني.
6. I feel safe using my voice to share my feelings and ideas.											٦. انا اشعر بالاطمئنان عندما استعمل صوتي للمشاركة باراي ومشاعري.
7. I am not ashamed to speak.											٧. انا لا اشعر بالخجل عندما أتكلم.
8. I feel my voice has the power to make a positive impact for my life and others.											٨. صوتي له القوة للتأثير ايجابيا على حياتي وعلي حياة الأخرين.
9. I listen to and consider what other people say.											٩.انا استمع الي الاخرين واخذ اراءهم في الحسبان.
10. I use my voice to share my feelings and ideas.											١٠. انا استعمل صوتي للتعبير عن مشاعري وافكاري.
11. I use my voice to help myself and others.											١١.انا استعمل صوتي لمساعدة نفسي والاخرين.
12. I use my voice with responsibility and courage.											١٢. انا استعمل صوتي بمسئولية وشجاعة.

هل هناك المزيد الذي تودي قوله عن صوتك؟ يمكنك اضافته بالخلف!

Is there anything else you would like to share about your voice? Write it on the back!

Mark an X on the line that corresponds with how you feel about your voice:
A. I am heard and understood at my home
B. I am heard and understood at my school
C. I am heard and understood in public

ضع علامة علي السطر المناسب كما تشعرين:
أ. إن صوتي مسموع و مفهوم في منزلي
ب. إن صوتي مسموع و مفهوم في مدرستي
ت. إن صوتي مسموع و مفهوم في الشارع

A / أ

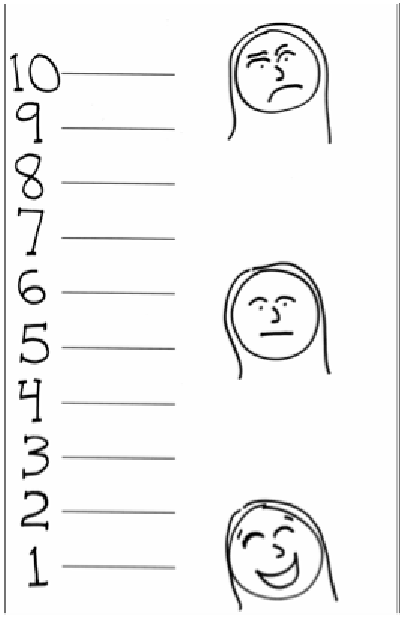

B / ب

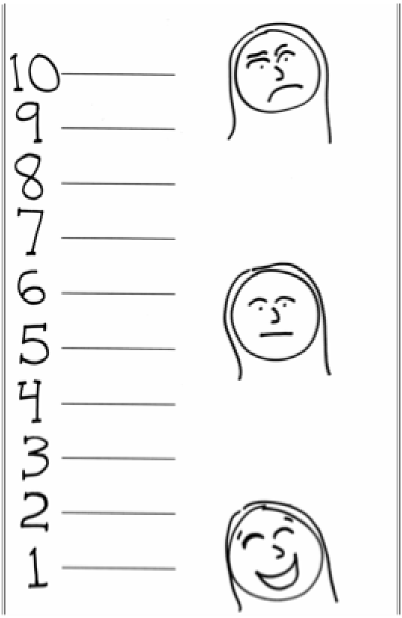

C / ت

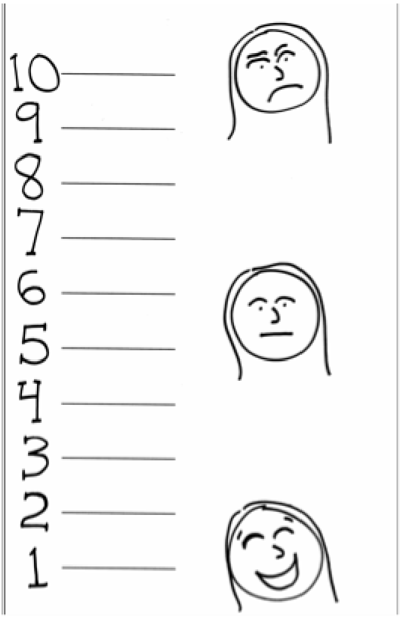

SPEAK.WORLD
Intellectual property of SPEAK
