# Supplementary material for: The effects of theatre-based vocal empowerment on young Egyptian women’s vocal and language characteristics
Source: PLoS One. 2021 Dec 31;16(12):e0261294. doi: 10.1371/journal.pone.0261294 (PMC8719750; doi:10.1371/journal.pone.0261294)
Supplement: S2 Appendix — (PDF) [file pone.0261294.s002.pdf]

يواجه الانسان في مراحل حياته عقبات كثيره؛ عليه ان لا يستسلم لها وهو يتطلع الي المستقبل ويحلم بالنجاح. فالنجاح هدف الانسان ولكن الوصول الي هذا الهدف يحتاج الي مجموعه عناصر تعينه على مواجهه الحياة وتساعده على الوصول الي النجاح. ومن بين تلك العناصر الثقة بالنفس والثقة في استخدام صوتك للتعبير عن نفسك لتحقيق أهدافك.
