# Supplementary material for: The effects of theatre-based vocal empowerment on young Egyptian women’s vocal and language characteristics
Source: PLoS One. 2021 Dec 31;16(12):e0261294. doi: 10.1371/journal.pone.0261294 (PMC8719750; doi:10.1371/journal.pone.0261294)
Supplement: S3 Appendix — (PDF) [file pone.0261294.s003.pdf]

Once there were four children whose names were Farida, Youssef, Sherif and Hadia. This story is about something that happened to them when they were sent away from Alexandria during the summer because it was the end of the school year.
